# The Educative Work of the School Doctor

**Published:** 1915-07-17

**Authors:** 


### THE EDUCATIVE WORK OF THE SCHOOL DOCTOR.*

### Nasal Troubles in School Children.

The most common complaint the school doctor sees in his periodical visits to schools is undoubtedly catarrhal rhinitis—the common cold. It is usually looked upon by teachers and parents alike as a very minor complaint, scarcely worth notice. Yet, as most of us know, it is a very potent factor in the causation of more serious defects. One child in the infant department suffering from it will easily infect a dozen other children, and occasion a rich crop of bronchitis and minor bronchial troubles that may lead to far graver issues. The attention of teachers and parents should, therefore, be drawn to these cases of colds in the nose and head. It should be made clear to all concerned that the common cold is a serious matter which should not be disregarded, but treated as soon as possible. Perhaps it is always best to exclude children suffering from it, but in the case of young children the parents are averse from accepting exclusion because the child is a trouble at home, while at school they are exempt from the obligation to look after it for some hours every day. In any case such children should be isolated in school; they should be given a separate seat, apart from the rest of the class, and should not be allowed to mingle with the other children in the playground until their colds are cured. Properly and quickly dealt with, the common cold in a child of school-going age is quickly cured; neglected, it invariably in young children leads to bronchial catarrh, which as quickly develops into peribronchial inflammation and lobular pneumonia. Where the condition is at all serious and the child has a flushed face with copious catarrhal discharge, the school doctor should insist upon exclusion and the child should be sent home, put to bed, and promptly treated.

Chronic catarrhal rhinitis is usually the result of a neglected cold in children who suffer from nasal obstruction due to adenoids, enlarged turbinals, or deflected septa. The cure here consists in removing the cause, and operative treatment is generally necessary before a permanently good result is obtained. The condition of the adenoid-suffering child is easily diagnosed, but it is sometimes overlooked that a relatively small patch of adenoids, leathery and hard, may give rise to nasal and ear trouble, and yet show none of the evidence of the existence of adenoids on which reliance is usually placed. It need not be recalled to mind that sepsis is of far greater importance in the adenoid condition than mere hypertrophy. The latter can be treated by syringing and breathing exercises, with usually satisfactory results if the home treatment is systematically and thoroughly carried out; the latter is only amenable to proper operative treatment. Where the mucosa of the nose is extensively involved, operative treatment must be thorough and in the hands of a specialist. The removal of hypertrophied turbinals is not the insignificant operation it is usually considered to be; the actual operative procedure is easy enough, but the after and preliminary treatment is highly important, and must be skilfully and thoroughly carried out.

Cases of deflected nasal septa are difficult to deal with. An operation is contraindicated where the child is below twelve years of age, and palliative treatment must be resorted to. In children above that age operation holds out fair chances of success, but it must be very skilfully done, otherwise there is a chance of the face being permanently disfigured by the sinking of the bridge of the nose. So long as the blocked nostril is not entirely obstructed operation may be postponed until a later age, preferably when the child has reached the age of sixteen or seventeen. Care should be taken in these cases that the obstructive adenoids are removed, and that the nostrils are periodically syringed by snuffing up a mild antiseptic solution such as salt and water or highly dilute Condy's fluid. Where there is much turgescence of the nasal mucosa a drop of adrenalin solution added to the fluid does good service, and the relief experienced after snuffing such a mixture up the nostrils is sometimes very evident.

Epistaxis is frequently met with among school children, and the doctor's attention is invariably drawn to it by the requests of teachers or nurses to treat cases of nose bleeding. In all cases the child should be carefully examined, for in the majority of cases epistaxis is merely a sign of a graver condition. It may precede the onset of acute febrile disorder, such as rheumatic fever, mumps, scarlet fever, or measles, or may be caused by increased blood pressure due to aortic or kidney disease, though both these are rare in school-going children. Yet their

* Previous articles appeared on June 19 and July 3, 1915.

rarity should not be taken as sufficient justification to exclude them without making a proper examination. The urine should be tested in all cases, and the nostrils carefully inspected with the aid of a speculum and good light, preferably that from an electric pocket lamp, whose beams can be thrown into the farthest recesses. Where a local condition is found to account for the bleeding the application of some styptic-adrenalin chloride solution or a solution of perchloride of iron will be found the most suitable, or simple plugging with dry gauze will stop the bleeding; old-fashioned household remedies, the key put down the child's back, for instance, will scarcely be sufficient, as they appear to be methods of suggestive treatment more than rational surgery. Where no styptic is available the application of cold, in the form of an ice or cold-water compress applied to the root of the nose, may be tried. Where epistaxis is frequent the child should be carefully examined, and will generally be found in need of simple tonic treatment. A daily dose of a few drops of Easton's or Huxley's syrup will generally suffice to cure the condition and prevent recurrence.

Atrophic rhinitis is usually easily diagnosed. It is fortunately a rare condition, met with in debilitated children, and may fairly be ascribed to neglected catarrhal conditions, though its etiology is not yet sufficiently elucidated to permit of a definite opinion on this point. Children suffering from it should be excluded from school, not only because they are an offence to the rest of the class, but also because the treatment necessary for a cure in this condition must be prolonged and thorough before any good result can be expected. Some of the cases met with are very intractable, and obstinately resist all treatment. In any case the child's general health must be strengthened by administering suitable tonics and good food. As many of these children are very poor such cases are suitable for the Care Committees' attention, and should be referred to them.

